# Integration of a Miniature Quartz Crystal Microbalance with a Microfluidic Chip for Amyloid Beta-Aβ_42_ Quantitation

**DOI:** 10.3390/s151025746

**Published:** 2015-10-12

**Authors:** Wenyan Tao, Qingji Xie, Hairui Wang, Shanming Ke, Peng Lin, Xierong Zeng

**Affiliations:** 1 Shenzhen Key Laboratory of Special Functional Materials & Shenzhen Engineering Laboratory for Advance Technology of Ceramics, College of Materials Science and Engineering, Shenzhen University, Shenzhen 518060, China; E-Mails: taowenyan2002@gmail.com (W.T.); wang8641@hotmail.com (H.W.); smke@szu.edu.cn (S.K.); 2 College of Electronic Science and Technology, Shenzhen University, Shenzhen 518060, China; 3 College of Chemistry and Chemical Engineering, Hunan Normal University, Changsha 410081, China; E-Mail: xieqj@hunnu.edu.cn

**Keywords:** integration, miniature quartz crystal microbalance, polydimethylsiloxane, amyloid polypeptide–Aβ_42_

## Abstract

A miniature quartz crystal microbalance (mQCM) was integrated with a polydimethylsiloxane (PDMS) microfluidic device for on-chip determination of amyloid polypeptide–Aβ_42_. The integration techniques included photolithography and plasma coupling. Aβ_42_ antibody was immobilized on the mQCM surface using a cross-linker method, and the resonance frequency of mQCM shifted negatively due to antibody-antigen binding. A linear range from 0.1 µM to 3.2 µM was achieved. By using matrix elimination buffer, *i.e.*, matrix phosphate buffer containing 500 µg/mL dextran and 0.5% Tween 20, Aβ_42_ could be successfully detected in the presence of 75% human serum. Additionally, high temperature treatments at 150 °C provided a valid method to recover mQCM, and PDMS-mQCM microfluidic device could be reused to some extent. Since the detectable Aβ_42_ concentration could be as low as 0.1 µM, which is close to cut-off value for Alzheimer patients, the PDMS-mQCM device could be applied in early Alzheimer’s disease diagnosis.

## 1. Introduction

Alzheimer’s disease (AD), a kind of irreversible progressive neurodegenerative disease, has been ranked as the 3rd killer of the elderly, only next to angiocardiopathy and cancer. Nowadays there are over 25 million cases of AD around the whole world, with about 5 million of these being found in China. Early prediction of AD is very important, since it is helpful to slow down the progression of the disease. Two amyloid polypeptides–Aβ_40_ and Aβ_42_, found in serum and cerebrospinal fluid, are proved to be the biomarkers of Alzheimer disease. The dominant mutation of amyloid polypeptide–Aβ_4_ protein precursor gene on normal chromosomes 21 and pressenilin-1 or 2 gene on chromosomes 14 is seen in AD patients before the age of 65. Amyloid precursor protein (APP), which belongs to the type I membrane glycoproteins, has at least 10 isomers arising from different splicing of 19 exons. The main transcripts are APP_695_, APP_751_ and APP_770_ [1,2,3]. The mechanism causing AD is still not clear. Research is concentrated on the structure of the neuron plaque and never fibers. The major component of neuron plaques is β-amyloid polypeptides (Aβ) generated from amyloid precursor protein (APP) by enzymatic cleavage involving β- and γ-secretase activities. Two kinds of Aβ configuration in neuron plaque are Aβ_40_ and Aβ_42_, which can be found in the serum and cerebrospinal fluid of healthy persons. The Aβ_40_ concentration in AD patients is equal to that observed in healthy persons. However, the Aβ_42_ concentration decreases significantly in AD patients, which reflects an increase of insoluble neuron plaque in the brain, therefore serum Aβ_42_ detection provides a valid method for early AD diagnosis. 

Existing detection technologies are divided into two categories: one is traditional molecular biology techniques such as enzyme-linked immunosorbent assay [4,5], electrochemical analysis [6,7,8,9], and liquid chromatography-mass spectroscopy technology [10]; another is frontier techniques such as surface plasma resonance, dot imprinting immunology and resonance light-scattering analysis [11,12]. Yu [9] proposed a method for highly sensitive determination of soluble β-amyloid peptides (Aβ_42_) that employed a detection bioconjugate of HRP-Au-gelsolin as an electrochemical nanoprobe. Aβ_42_ was captured onto the electrode surface due to its specific binding to surface-confined gelsolin. Because 3,3,5,5-tetramethylbenzidine could be specifically catalyzed at 0.35V in the presence of H_2_O_2_ by HRP labeled on Au nanoparticles, this would produce a signal related to the amount of surface-confined HRP, thus realizing the detection of Aβ_42_ specifically. The proposed methodology displayed satisfactory sensitivity, with a detection limit down to 28 pM. Clarke [10] successfully separated serum β-amyloid peptides in several minutes through an Atlantics C_8_ chromatographic column, and detected the amount of endogenous Aβ_42_ through combination with mass spectroscopy. The limit of detection was 0.2 pM. Yu [12] detected β-amyloid peptides by the fluorescence signal increase at 463 nm of a new hybrid fluorescent probe–Fe_3_O_4_@Au upon reaction with β-amyloid peptides. The linear range is from 5.0 fM to 5.56 pM, with a detection limit down to 1.2 fM. Although they offer advantages of high-throughput, high-sensitivity and low detection limits, these methodologies usually involve arduous manual procedures, long incubation times, large sample consumption and photo-bleaching during fluorescence detection, which are not conducive to portable applications. Meanwhile, electrochemical technology and immunoassay have other shortcomings such as expensive bioprobes, tedious labeling steps, side reactions, and non-specific binding. Therefore, it is urgent to develop a low-cost and portable form technique for the determination of β-amyloid peptide.

Microfluidic-based approaches allow rapid and high-throughput analyses with less sample consumption, which make them very attractive for point-of-care diagnosis [13,14]. Existing detection techniques combined with microfluidic devices include electrochemical immunoassay [15], fluorescence methods [16] and surface-enhanced Raman scattering-based immunoassay [17]. These methods are not suitable for point-of-care diagnostics of peptides and proteins in human serum because they entail long response times, expensive instruments, and tedious labeling processes. The quartz crystal microbalance (QCM) is a type of mass sensitive transducer that has been extensively applied in the detection of proteins [18], antibodies [19], cancer biomarkers [20] and organic substances [21]. It works in a label-free fashion since the resonance frequency shift caused by mass loading on the surface condition can be measured directly. Few papers have reported the integration of miniature QCMs with microfluidic systems. A miniature QCM with a quartz diameter of 3 mm fixed on a PDMS flow cell for detecting protein A from *Staphylococcus aureus* in liquid phase was reported [22]. To reduce the boundary diffusion layer and total sample consumption, a miniaturized flow reaction chamber with a volume of 3.5 µL was developed for a quartz crystal microbalance with dissipation monitoring [23]. Meanwhile, microfluidic devices based on polydimethylsiloxane (PDMS) possess the advantages of low cost, rapid fabrication and excellent biocompatibility compared to those on quartz, silicon or glass. Hence, in this work, a 13 × 10^6^ Hz miniature QCM resonator (mQCM) with a diameter of 6 mm was integrated with a PDMS microfluidic device for on-chip determination of amyloid polypeptide–Aβ_42_. The integration process, serum sample analysis and device reusability were investigated in detail. 

## 2. Experimental

### 2.1. Principle of MQCM in a Liquid Microfluidic System

**Figure 1:**
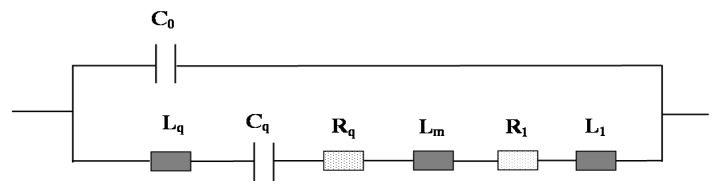
Equivalent circuit of a rigid layer-modified mQCM in liquid phase.

According to the Sauerbrey equation, the shift in resonance frequency (Δ*f_0_*) resulting from a mass change (Δ*m*) on the mQCM surface can be expressed as follows:

(1) $$\text{Δ}f_{0}=-\frac{2{f_{0}}^{2}}{A\sqrt{{\text{ρ}}_{\text{q}}{µ}_{\text{q}}}}\text{Δ}m$$

where *f*_0_ is the resonance frequency of bare mQCM, *A* is the piezoelectric active crystal area; µ_q_ and ρ_q_ are the elastic modulus and density of quartz, respectively. Figure 1 shows the equivalent circuit of a coated mQCM in liquid phase. The motional inductance (*L*_q_) relates to the mechanical vibration inertia of quartz vibration process. The motional capacitance (*C*_q_) corresponds to the mechanical elasticity and motional resistance *R*_q_ equivalent to the energy losses in the vibration process. The static capacity (*C*_0_) is formed from dielectric quartz substrate between the two electrodes when there is no vibration. Its value is related to the geometric size of the quartz and electrode area.

When only one side of the quartz is in contact with liquid, an additional inductance (*L*_1_) and resistance (*R*_1_) will be formed by liquid loading. Their values are related to the mass, viscosity and density properties of the vibrating liquid. A thin rigid layer is commonly coated on mQCM sensor for specific applications, which brings an additional inductance by mass loading, named as *L*_m_, as shown in Figure 1.

An equation reflecting liquid loading effects for our case can be derived from Martin’s equations [24] and the equation in reference [25,26]:

(2) $$\Delta R_{t}=2\pi f\Delta L_{t}\approx -4\pi L_{\text{qa}}\Delta f_{\;0}\;$$

where Δ*R*_t_ and Δ*L*_t_ are the changes in total resistance and total inductance, respectively. *L*_qa_ is the motional inductance for mQCM sensor in air. *f*_0_ can be used approximately in the calculation instead of *f* with an error below *ca.* 0.3%, because the *f* value is hard to obtain. Accordingly, if the experimental responses of Δ*R*_t_, Δ*f*_0_ and Δ*L*_t_ satisfy Equation (2), then the frequency shift (Δ*f*_0_) should be governed by variation in the density and viscosity of local solution near mQCM sensor surface. Otherwise, mass change is the effective factor [27]. 

In real data analysis, Δ*R*_t_ and Δ*L*_t_ values can be calculated from following equations, which could be derived from the equations in reference [26]:

(3) $$G=\frac{G_{max}}{2}=\frac{1}{2R_{\text{t}}}\;$$

(4) $$\Delta f_{G_{1/2}}=\frac{R_{\text{t}}}{2\pi L_{\text{t}}}$$

where *G*_max_ is maximum of conductance value; *R*_t_ and *L*_t_ are total resistance and total inductance, respectively. $\Delta f_{G_{1/2}}$ is the full width at half maximum of the conductance spectrum. Therefore, we will investigate in detail if there is any difference in the half bandwidth of the conductance spectrum between the bare and the modified mQCM sensor.

### 2.2. Chemicals and Reagents

3-Mercaptopropionic acid (3-MPA, ≥99.0%), *N*-hydroxysuccinimide (NHS, 98.0%), *N*-(3-dimethylaminopropyl)-*N*'-ethylcarbodiimide hydrochloride (EDC, ≥99.0%), bovine serum albumin (BSA, ≥97.0%) and human serum (from human male AB plasma) were purchased from Sigma-Aldrich (St. Louis, MO, USA). Monoclonal amyloid polypeptide Aβ_42_ antibody and Aβ_42_ were ordered from Zi-Yu Biotech Co. Ltd. (Shanghai, China). The Sylgard 184 silicon elastomer kit was purchased from Dow Corning (Midland, MI, USA). All chemicals were of analytical grade and used without further purification.

### 2.3. Fabrication of PDMS-mQCM Microfluidic Device

**Figure 2:**
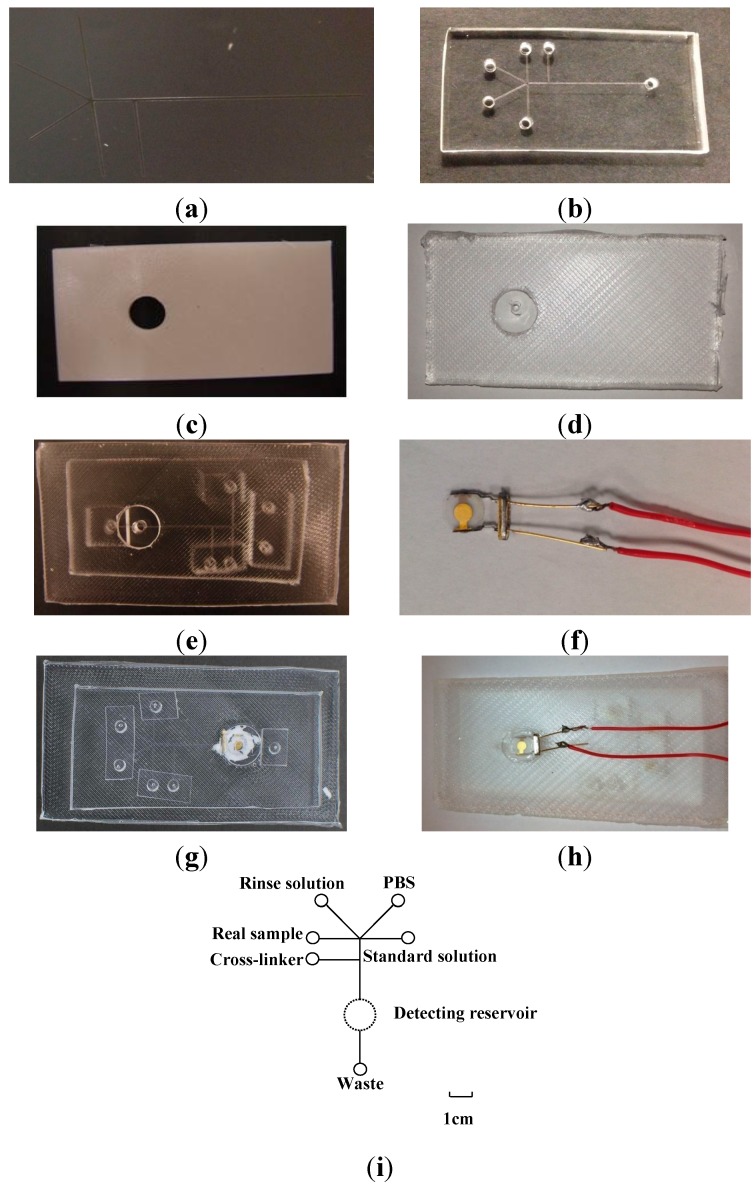
Photographs of (**a**) Si mold; (**b**) ABS mold; (**c**) PDMS microfluidic chip; (**d**) mounting PDMS slab; (**e**) PDMS-PDMS microfluidic device; (**f**) mQCM; (**g**) front-side and (**h**) backside of PDMS-mQCM microfluidic system; (**i**) Layout of an integrated PDMS-mQCM microfluidic system; standard solution: from 0.025 µM to 5.2 µM Aβ_42_. Scale bar: 1 cm.

The detailed fabrication process is illustrated in Figure 2.

#### 2.3.1. Si Mold

The silicon-mold fabrication follows standard photolithography procedures. Negative photoresist SU-8 2035 was spin-coated on N-type silicon (Si <100>) wafer surface at a speed of 1000 rpm for 40 s. The wafer was soft-baked at 65 °C for 3 min and then 95 °C for 9 min; and followed by exposure to UV light for 3 min together with the mask. After exposure, it underwent post-baking at 65 °C for 2 min and then at 95 °C for 7 min. The development lasted for 10 min using SU-8 developer. SU-8 with 100 µm width and 40 µm height was patterned on Si wafer, named as Si mold, as shown in Figure 2a. Si mold was prepared for imprinting PDMS microfluidic chip.

#### 2.3.2. ABS Mold

Acrylonitrile butadiene styrene (ABS) is a thermoplastic polymer material which consists of acrylonitrile, butadiene and styrene monomers. A 40 mm × 70 mm ABS mold plane with a 10 mm diameter through-hole was fabricated by a prototyping machine (*u*Print SE Plus, Chicago, IL, USA), as shown in Figure 2b.

#### 2.3.3. PDMS Microfluidic Chip and Mounting PDMS Slab Molding

Firstly, Si and ABS mold was cleaned with a mixture of detergent and 70% ethanol (v/v = 1:10), rinsed with water and dried with N_2_ gas. Secondly, a curing agent and silicon elastomer of PDMS prepolymer were mixed thoroughly at a weight ratio of 1:10. The prepolymer mixture was poured on Si mold and ABS mold, and then degassed in a vacuum chamber for 2 h. Finally, molds were put on a heater for polymerization at 70 °C overnight. After curing, PDMS replicas were peeled from the mold. Holes with diameters of 3 mm were punched in the PDMS microfluidic chip at the end of the micro-channels (shown in Figure 2c). Another 3 mm hole was punched on the PDMS slab (shown in Figure 2d) to match the diameter of the gold electrode on the mQCM and worked as a solution reservoir.

#### 2.3.4. PDMS-mQCM Microfluidic Device

Freshly prepared PDMS microfluidic chip and mounting PDMS slab were first treated by oxygen plasma and immediately brought in contact with each other, and finally baked at 80 °C for 2 h to achieve permanent bonding. To connect with tubing for solution injection, small PDMS pieces punched with 0.75 mm holes were also stuck to PDMS microfluidic chip by the oxygen plasma method. This PDMS-PDMS device is shown in Figure 2e. *AT-*cut quartz crystals with resonance frequency of 13 × 10^6^ Hz (Chen Jing Electronics, Beijing, China) were used as mQCM sensors in this work. The diameter of quartz crystal was 6 mm, and diameter of gold electrode was 3 mm. Copper wires were soldered to the two legs of gold electrodes on mQCM, as shown in Figure 2f. The mQCM was fixed to PDMS-PDMS microfluidic device using 704 silicone gels (Wuxi Adhesive Factory, Wuxi, China), which formed a PDMS-mQCM microfluidic device, as shown in Figure 2g (front side) and Figure 2h (backside). 

#### 2.3.5. Layout of PDMS-mQCM Microfluidic Device

Figure 2i shows the layout of the PDMS-mQCM microfluidic device. As shown in the layout, rinse solution, buffer, cross-linker, Aβ_42_ standard solution and real sample are injected into the device from different reservoirs to avoid contamination. The total size of the PDMS-mQCM microfluidic device is around 50 mm (W) × 80 mm (L) × 6 mm (H). 

### 2.4. Immobilization of Aβ_42_ Antibody 

**Figure 3:**
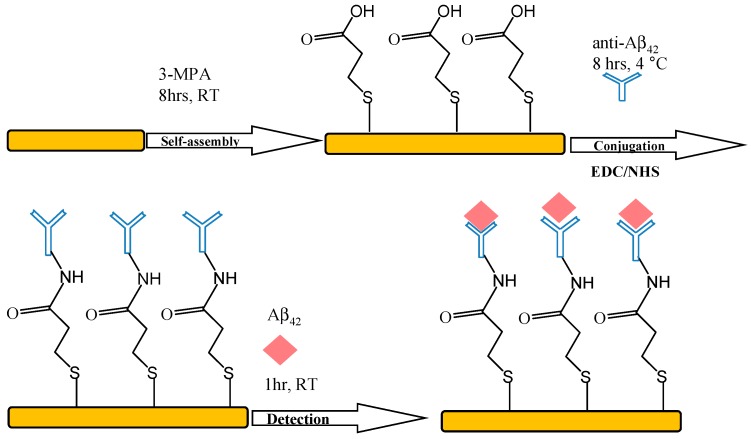
Schematic representation of the immobilization process for Aβ_42_ antibody and detection of Aβ_42_.

A cross-linker method was adapted for Aβ_42_ antibody immobilization. Five mM 3-MPA solution was injected into the detection reservoir of the PDMS-mQCM microfluidic device for 8 h. Then the reservoir was rinsed with ethanol and water. Next 20 mM phosphate buffer solution (PBS, pH 7.0) containing 24 mg/mL EDC and 6 mg/mL NHS was injected into the reservoir and incubated for 1 h. The channels and reservoirs were rinsed with PBS afterwards. 2 µM Aβ_42_ antibody in PBS was then injected into the reservoir and incubated for 12 h at 4 °C, and rinsed with PBS thoroughly to remove the absorbed. Then the defective sites were blocked with 10 µM BSA in PBS. A schematic representation of these processes is shown in Figure 3.

### 2.5. Measurements 

Aβ_42_ can be extracted from blood serum and directly injected into the real sample reservoir of the PDMS-mQCM microfluidic device, and then rapidly determined by an Aβ_42_ antibody-modified mQCM sensor using the piezoelectric quartz crystal impedance technique (PQCI). Injection mode was adapted. All on-chip fluid flow was controlled via a network of syringes, valves and reservoirs. Syringes were connected to the inlet of PDMS-mQCM microfluidic device by using Teflon tubing for injecting specific volumes of solution. The resonance frequency response and other equivalent circuit parameters of the mQCM sensor during the antigen-antibody binding process were determined by the PQCI technique. Frequency response was measured by an impedance network analyzer (E5016B, Agilent Technology, Santa Clara, CA, USA). The conductance (*G*) and susceptance (*B*) of the sensor were also measured synchronously by an impedance test. Automatic data acquisition was achieved by a program written in Visual Basic 6.5 (VB 6.5). Impedance measurements were conducted under conditions of 1601 points, an IF bandwidth value of 5000 Hz, and a source power of 0 dBm. Acquired data was analyzed by a home-written Matlab program to extract the maximum *G* value and corresponding resonance frequency. All measurements were performed at a stable temperature of 25 ± 1 °C.

## 3. Results and Discussion

### 3.1. On-Chip Determination of Aβ_42_

**Figure 4:**
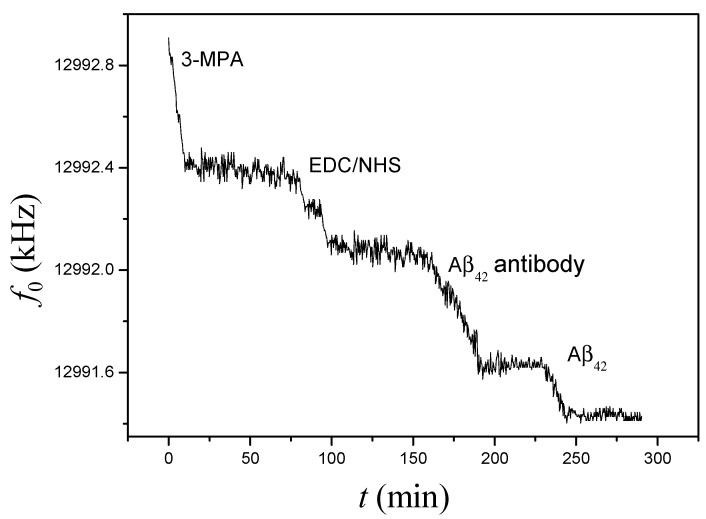
On-line monitoring frequency response for a complete sequence of immobilization of Aβ_42_ antibody and detection of Aβ_42_ on PDMS-mQCM microfluidic system.

Figure 4 shows the frequency response of the immobilization processes for Aβ_42_ antibody and Aβ_42_ on the PDMS-mQCM microfluidic device using a cross-linker method. Frequency shifts were observed for each step: −550.2 Hz for 3-MPA, −353.0 Hz for EDC and −443.0 Hz for Aβ_42_ antibody. The response frequency shift towards 0.8 µM Aβ_42_ was −195.6 Hz on the Aβ_42_ antibody-modified PDMS-mQCM microfluidic device. 

**Table 1:** Comparison of equivalent circuit parameters between on the bare and those on modified mQCM in liquid phase.

Device	*G*_max_ (mS)	*R*_t_ (Ω)	Δ*R*_t_ (Ω)	Δ*f*_0_ (Hz)	*K*
PDMS-mQCM	3.79 ± 0.02	504.5 ± 3.5	NA	NA	NA
Aβ_42_ antibody-PDMS-mQCM	1.23 ± 0.05	860.6 ± 5.3	356.1 ± 0.6	−1528 ± 3	2.94 ± 0.03
Aβ_42_-Aβ_42_ antibody-PDMS-mQCM	1.20 ± 0.03	863.8 ± 6.8	3.2 ± 0.3	−218 ± 1	46.74 ± 0.50

NA: not applicable; $K=\frac{-4\pi L_{\text{qa}}\Delta f_{0}}{\Delta R_{\text{t}}}$; *L*_qa_ is the motional inductance for mQCM sensor in air.

The frequency response for 3-MPA became stable after 75 min, which indicated that adsorption of –SH function groups on the mQCM surface approached saturation. Similar phenomena could be observed during EDC and Aβ_42_ antibody immobilization with 62 min and 70 min saturation times, respectively. Meanwhile, the conductance spectrum data during immobilization processes for Aβ_42_ antibody on the mQCM were analyzed. The calculated *G*_max_, *R*_t_, *f*_0_, Δ*R*_t_ and Δ*f*_0_ values are given in Table 1. The *K* value was set for evaluation of the experimental responses (Δ*R*_t_ and Δ*f*_0_) satisfying Equation (4) or not. *L*_qa_, the motional inductance for mQCM sensor in air, is 54.6 mH, which calculated from the conductance spectra of bare-mQCM sensor in air. As seen in Table 1, *K* values are above 1, so mass loading are an effective factor during the antibody immobilization step and the viscosity-density effect of the solution near the interface can be negligible.

### 3.2. Calibration Curve

**Figure 5:**
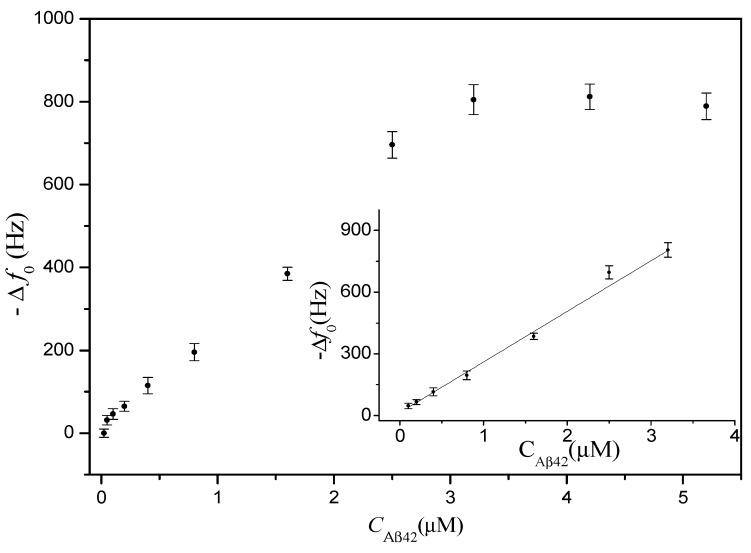
The response frequency shift for on-chip determination of different concentrations of Aβ_42_ in PBS. Inset figure is a calibration curve of Aβ_42_.

Determination of Aβ_42_ in PBS with different concentrations from 0.025 µM to 5.2 µM was conducted on the PDMS-mQCM microfluidic device. Results are shown in Figure 5. A linear range from 0.1 µM to 3.2 µM was found. The regression equation was obtained as follows: Δ*f*_0_(Hz) = −246.15 × *C*_Aβ42_(nM) − 14.44, with a correlation coefficient of 0.991. 

### 3.3. Real Sample Assay

#### 3.3.1. Non-Specific Binding Assay

Detection of biomarkers in real samples is commonly conducted in spiked buffer solutions containing human serum up to 25% (v/v) [28,29], which may be too low for clinically relevant applications. Therefore, to validate this assay in human serum, we selected a buffer solution containing a serum concentration of 75% (v/v) as spiked buffer. Meanwhile, to eliminate the non-specific serum protein binding to the mQCM sensor surface, some desorption reagents were added in PBS. A new PBS matrix buffer containing 200 µg/mL BSA, 0.5 M NaCl, 500 µg/mL dextran and 0.5% Tween 20 was used to dilute the serum. The non-specific binding of serum protein in PBS matrix on the Aβ_42_ antibody-modified mQCM sensor was evaluated and compared with other conditions. Results are listed in Table 2. Obviously, there are −36.5 Hz and −89.5 Hz responses for 25% serum and 75% serum, respectively, on a bare gold-QCM sensor, while on the Aβ_42_ antibody-modified mQCM sensor, there are almost no responses for both 25% and 75% serum. A possible reason is as follows: there are various enzymes, peptides and proteins in human serum, such as glutamic-pyruvic transaminase and albumin. Non-specific binding adsorption of enzymes or proteins in human serum occurred on the bare gold-mQCM sensor surface. The adsorption of enzymes or proteins on a bare gold-mQCM surface is similar to that of thiol-alcohol compound on the gold surface due to the formation of Au-S bonds between the Cys34 group in enzymes or proteins and the gold surface [30], while on the Aβ_42_ antibody-modified mQCM sensor, the gold surface was occupied by the antibody and BSA layer, so Au-S bonds could not formed by enzymes or proteins in the serum. 

**Table 2:** Non-specific binding determination for different assays.

Device	Injected Sample ^a^	Δ*f*_0_ (Hz)
PDMS-mQCM	25% serum	−36.5 ± 3.0
PDMS-mQCM	75% serum	−89.5 ± 7.8
Aβ_42_ antibody-PDMS-mQCM	25% serum	0
Aβ_42_ antibody-PDMS-mQCM	75% serum	0
Aβ_42_ antibody-PDMS-mQCM	0.8 µM Aβ_42_ + 75% serum	−189.7 ± 8.6
Aβ_42_ antibody-PDMS-mQCM	1.6 µM Aβ_42_ + 75% serum	−356.0 ± 9.7

^a^ Injected samples were diluted with PBS with additives: 200 µg/mL BSA, 0.5 M NaCl, 500 µg/mL dextran and 0.5% Tween 20.

#### 3.3.2. Detection of Aβ_42_ in 75% Human Serum

**Figure 6:**
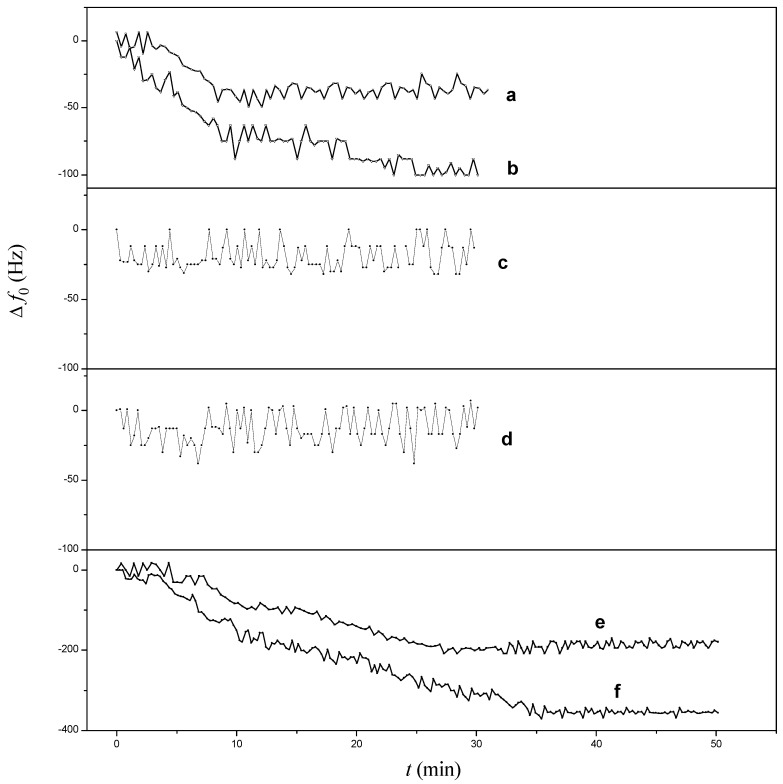
Continuous monitoring frequency response for different devices in different samples: (**a**) PDMS-mQCM in 25% serum; (**b**) PDMS-mQCM in 75% serum; (**c**) Aβ_42_ antibody-PDMS-mQCM in 25% serum; (**d**) Aβ_42_ antibody-PDMS-mQCM in 75% serum; (**e**) Aβ_42_ antibody-PDMS-mQCM in 0.8 µM Aβ_42_ and 75% serum; (**f**) Aβ_42_ antibody-PDMS-mQCM in 1.6 µM Aβ_42_ and 75% serum.

To validate the analytical reliability of our PDMS-mQCM microfluidic device in real samples, two different concentrations of Aβ_42_ spiked in 75% (v/v) human serum with PBS-matrix buffer were selected for testing. There is a −189.7 Hz response for 0.8 µM Aβ_42_ and −356.0 Hz for 1.6 µM Aβ_42_ (seen in Table 2 and Figure 6). These values are close to the responses in pure PBS (−195.6 Hz and −385.0 Hz, as seen in Figure 5). This proved that desorption reagents may eliminate serum protein non-specific binding to the Aβ_42_ antibody on the sensor surface. On the other hand, the measured frequency of Aβ_42_ antibody-PDMS-mQCM with 0.8 μM or 1.6 μM Aβ_42_ in 75% human serum do not agree with those in pure PBS (according to the regression equation). A reasonable explanation is as follows: antigen-antibody reactions at the solid-liquid interface are diffusion limited due to depletion of reactants close to the surface [31]. The target diffusion rate would be related to the physical properties of the environmental buffer, such as viscosity and conductivity and so on. Therefore, results would be different in different testing environments, even for the same antibody-modified sensor. Additionally, lower serum Aβ_42_ concentrations (below 50 nM) cannot be detected at present, limited by the system sensitivity and small volume injection. We can detect low serum Aβ_42_ concentrations through pre-concentration methods in clinic analysis. The aim of this work is to provide an integrated PDMS-mQCM microfluidic device as a primary product for a portable microfluidic sensor. For clinic applications, the E5016B impedance network analyzer could be replaced by a simple chip, such as an AD9854 DDS chip, and hence the PDMS-mQCM microfluidic device could be considered as a fast-response, low-cost and portable sensor for determination of Aβ_42_.

### 3.4. Reusability

The reusability of the PDMS-mQCM microfluidic device is crucial for sensor cost. It was evaluated by measuring the resonance performance of the sensor before and after removing modified layers. A heating method was used for removing modified layers. Chandekar *et al.* investigated thermal stability of alkanethiol self-assembled monolayer (SAM) film adsorbed on a gold surface [32]. Their results indicated that Au-S bonds started to rupture at 110 °C, whereas physical adsorption of SAM film on the gold surface remained strong. Au-S bonds might form again and SAM film would adsorb on the surface. If a temperature of 150 °C was applied, almost all Au-S bonds would break and physical adsorption would become very weak. The Au-S bond cannot form again at 150 °C. In this work, the used PDMS-mQCM microfluidic system was heated to 150 °C over 60 min, then allowed to cool down to room temperature, and rinsed with ethanol and water prior to measurement. Table 3 shows the effect of high temperature on the conductance of PDMS-mQCM in the liquid phase. After heat treatment, a conspicuous positive frequency shift of 1470.0 Hz and conductance shift of 1.62 mS can be observed for the Aβ_42_ antibody-modified mQCM. The resonance frequency and conductance increases of the used and modified mQCM were close to those of bare mQCM. This indicated that the MPA-Aβ_42_ antibody layer had been almost completely removed by the high temperature treatment due to Au-S bond breaking. On the other hand, the resonance frequency was different from that on a bare one. The possible reason could be that AuO was formed on the mQCM surface due to the high temperature treatment. Hence, the high temperature treatment provides a valid method to recover the mQCM surface, and make the proposed PDMS-mQCM microfluidic device reusable.

**Table 3:** Effect of high temperature on the conductance of the PDMS-mQCM in liquid phase.

Device	*f*_0_ (Hz)	SD (Hz)	*G*_max_ (mS)	Δ*f*_0_ (Hz)	Δ*G*_max_ (mS)
PDMS-mQCM	12.99275 × 10^6^	3	3.90 ± 0.07	NA	NA
Aβ_42_ antibody-PDMS-mQCM ^a^	12.992675 × 10^6^	5	3.00 ± 0.03	75 ± 3	0.90 ± 0.01
Aβ_42_ antibody-PDMS-mQCM ^b^	12.991205 × 10^6^	9	1.38 ± 0.02	1470 ± 8	1.62 ± 0.02

NA: not applicable; ^a^ after 150 °C heating treatment; ^b^ before heating treatment.

## 4. Conclusions

In this work, a miniature QCM sensor was successfully integrated with a PDMS microfluidic chip, which was used in on-chip determination of an Alzheimer disease’s biomarker −Aβ_42_. The linear range was from 0.1 µM to 3.2 µM. By using a matrix elimination buffer containing 200 µg/mL BSA, 0.5 M NaCl, 500 µg/mL dextran and 0.5% Tween 20, Aβ_42_ could be successfully detected in a high serum concentration of 75%. Meanwhile, high temperature treatment with 150 °C could recover the modified mQCM surface, which makes the PDMS-mQCM microfluidic system reusable. Therefore, the prposed PDMS-mQCM microfluidic device is reusable, has fast-response and small-sample consumption, and is applicable for early Alzheimer disease diagnosis in the clinic.
